# In Vitro Evaluation of Leakage at Implant-Abutment Connection of Three Implant Systems Having the Same Prosthetic Interface Using Rhodamine B

**DOI:** 10.1155/2014/351263

**Published:** 2014-05-11

**Authors:** Antoine Berberi, Georges Tehini, Khaldoun Rifai, Farah Bou Nasser Eddine, Nabil El Zein, Bassam Badran, Haidar Akl

**Affiliations:** ^1^School of Dentistry, Lebanese University, P.O. Box 4, Hadath, Lebanon; ^2^Ecole Doctorale, PRASE, Lebanese University, P.O. Box 4, Hadath, Lebanon

## Abstract

*Objectives*. Hollow space between implant and abutment may act as reservoir for commensal and/or pathogenic bacteria representing a potential source of tissue inflammation. Microbial colonization of the interfacial gap may ultimately lead to infection and bone resorption. Using Rhodamine B, a sensitive fluorescent tracer dye, we aim in this study to investigate leakage at implant-abutment connection of three implant systems having the same prosthetic interface. *Materials and Methods*. Twenty-one implants (seven Astra Tech, seven Euroteknika, and seven Dentium) with the same prosthetic interface were connected to their original abutments, according to the manufacturers' recommendation. After determination of the inner volume of each implant systems, the kinetic quantification of leakage was evaluated for each group using Rhodamine B (10^−2^ M). For each group, spectrophotometric analysis was performed to detect leakage with a fluorescence spectrophotometer at 1 h (T0) and 48 h (T1) of incubation time at room temperature. *Results*. Astra Tech had the highest inner volume (6.8 **μ**L), compared to Dentium (4 **μ**L) and Euroteknika (2.9 **μ**L). At T0 and T1, respectively, the leakage volume and percentage of each system were as follows: Astra Tech 0.043 **μ**L or 1.48% (SD 0.0022), 0.08 **μ**L or 5.56% (SD 0.0074), Euroteknika 0.09 **μ**L or 6.93% (SD 0.0913), 0.21 **μ**L or 20.55% (SD 0.0035), and Dentium 0.07 **μ**L or 4.6% (SD 0.0029), 0.12 **μ**L or 10.47% (SD 0.0072). *Conclusion*. The tested internal conical implant-abutment connections appear to be unable to prevent leakage. In average, Astra Tech implants showed the highest inner volume and the least leakage.

## 1. Introduction

In two-stage implant therapy, screwing the abutment to the implant results in a gap between components. The implant-abutment gap, or inner space, acts as a bacterial reservoir having a degree of communication with the oral cavity, which could interfere with peri-implant tissue health and function [1–3].

Several adverse mechanical and biological consequences may occur.

Mechanical complications such as increased incidence of abutment rotation [4–8], screw loosening [9, 10], and preload reduction [11] have been reported to occur with poorly adapted abutments.

Biological complications such as mucositis [12] and bone loss [13, 14] have also been reported. In most implant systems, bidirectional exchange of fluids takes place at the level of the alveolar bone crest and is considered to be an important factor for chronic inflammatory infiltration and marginal bone loss [1–3, 14–16]. Especially during function and under occlusal loading, micromovement between abutment and implant will create volumetrically variation in the inner space of the implant system [17–19].

Several investigators aimed to quantify bacterial leakage of different implant systems. These studies investigated corpuscular bacterial leakage [20–24] or small molecules like endotoxin, [25–27], toluidine blue [28], and gas flow [29, 30].

Different amounts of suspension have been used to inoculate the implants in microleakage studies; amounts range from 0.3 *μ*L to 5 *μ*L [20, 21, 24, 25, 28, 31].

The inner volume and gap seem to be specific for each implant systems. Berberi et al. [32] showed that the inner volume was related to the connection design and to the type of inoculating solution. So for a specific solution and to avoid under- or overflow during leakage measurement, the internal volume must be evaluated before implant-abutment assembly.

Therefore, the aim of this study is

1. to determine the inner volume of three implant systems having the same prosthetic interface;
2. to test in vitro, the leakage of those three implant systems using a highly sensitive fluorescent tracer dye the Rhodamine B.

The hypothesis of the present study is that implant systems with the same prosthetic interface have the same inner volume and are similar regarding leakage.

## 2. Material and Methods

### 2.1. Implants and Abutments

Three implant systems (Astra Tech Implant System, Dentium and Euroteknika) were used in this study. The three implant systems used have the same internal implant-abutment connection interface. It is a conical-hex connection with 11° angulation. The restorative components are compatible in between systems and the prosthetic platform diameters are similar. In this study, items were all prefabricated and used as delivered by the manufacturers.

They were divided into 3 groups: 

*Group A*. seven OsseoSpeed implants (5 mm × 11 mm) connected to Ti Design abutments (5.5 mm, 1.5 mm), (Astra Tech Implant system, DENTSPLY Implants System, Mölndal, Sweden).

*Group B*. seven Natea implants (6 mm × 12 mm) connected to Natea abutments (5.8 mm, 2 mm), (Euroteknika Groupe, Sallanches, France). 

*Group C*. seven Implantium MF implants (4.8 mm × 12 mm) connected to dual abutments (5.5 mm, 1.5 mm), (Dentium Implant System, Seoul, North Korea).

### 2.2. Test Procedure

Rhodamine is used as fluorescence dye for biological assays. Rhodamine B is very soluble in water (~50 g/L) and fluoresces upon reaction with photogenerated oxyradicals. After an excitation at 535 nm wavelengths, the Rhodamine B-emitted fluorescence can easily be detected using spectrophotometer [33–35].

Rhodamine B (Rh B) (10^−2^) was prepared by dissolving 0.1 g of Rh B (Sigma R 6626-25G) in 20 mL of distilled water.

To appropriately quantify the amount of leakage of each implant-abutment combination, a calibration curve was determined using four different concentrations of Rh B (10^−7 ^M, 5 × 10^−7 ^M, 10^−6 ^M, and 2.5 × 10^−6 ^M). In all our experiments, we have used a wavelength monitoring mode of the VISION collect software to acquire the exact absorbance spectra. A special cuvette (Perkin Elmer Luminescence Spectroscopy Cells Part number B0631104) (3 measurements per concentration) was used for the fluorescence measurements. Calibration curve was determined by linear regression using GraphPad Prism 6.

The inner volume of each implant-abutment combination was evaluated as described in the pilot study [32].

**Figure 1:**
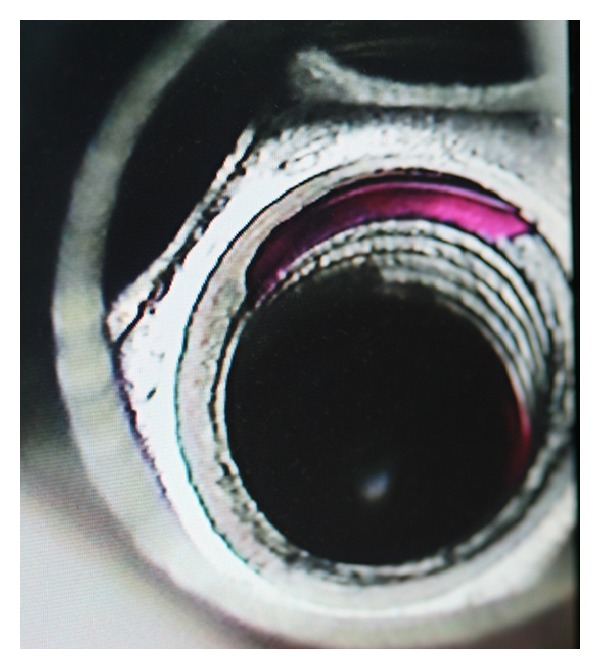
Placement of Rhodamine B solution inside the implant.

A single channel micropipette (L 322606, Pipetman, Gilson Service, France) was utilized to place 0.1 *μ*L of (10^−2 ^M) of the Rh B solution in the deepest portion of the internal compartment of the different combinations. A stereomicroscope was used to facilitate this procedure (Figure 1).

A vise connected to a bench was utilized to hold the implants in position in order to connect the abutments at the manufacturers' torque recommendations (25 N/cm for group A, 35 N/cm for group B, and 30 N/cm for group C).

**Figure 2:**
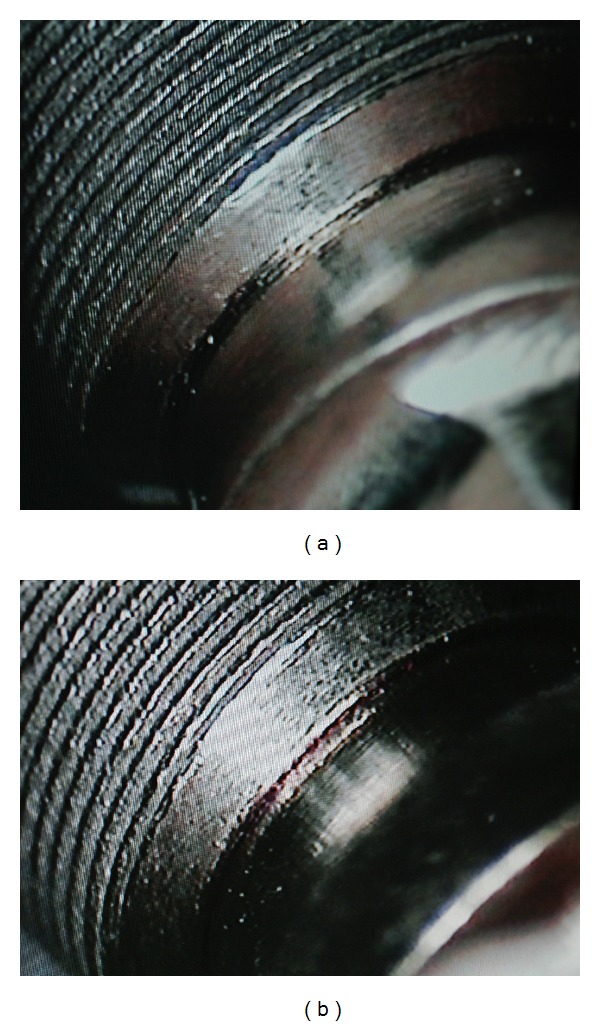
Picture of the assembly through the stereomicroscope with a 10x magnification (a) showing no leakage and (b) with leakage.

Leakage on implant-abutment interface (I-A-I) was accurately observed through the stereomicroscope with a full magnification of 10x (Figures 2(a) and 2(b)).

After each leak-measurement experiment, a clean-up process was conducted to displace any remaining trace of Rh B. For that, after each manipulation, implants, abutments and screws were rinsed many times with ethanol 70% and with distilled water successively using a vortex machine (Wisd Vortex Mixer DAIHAN Scientific Co., Korea) and then sterilized in an autoclave class B at 121° Celsius, at 1 kg/cm^2^ of pressure for 30 minutes (W&H Lisa Sterilizers, Sydenham, Christchurch, New Zealand) [36, 37].

The complete process was repeated many times by inoculating an increasing volume (by 0.1 *μ*L) of the (10^−2 ^M) Rh B solution, till reaching the maximal keeping capacities, volume with which we start to detect a leakage in each combination. Then, the seven implants in each combination group of implant abutments were used to confirm the volumes (keeping capacities) and the presence of leakage. The last volume that did not show any leakage is considered as the inner volume of the implant.

After calculation of the keeping capacities of all groups, the last volume with no leak (*x*) and the first volume showing a leak (*x* + 0.1 *μ*L) were used in the study to quantify the amount of leak between the different implant-abutment combinations.

Seeking an increased precision, we have used a controlled automated pipette (L322606, Pipetman, Gilson Service, France) together with single ultrathin tips (1310A, 236, Ranin, USA) to introduce the appropriate volume of Rh B solution in the deepest internal volume of the implant then the abutment was screwed according to the manufacturer's recommendations. To achieve the recommended torque levels, a screw connected to a bench was utilized to hold the implants in position.

**Figure 3:**
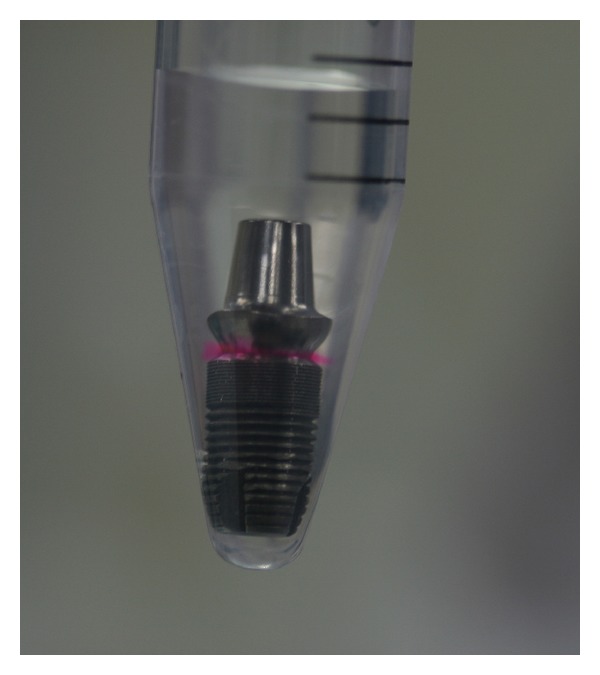
The assembly placed in vials filled with 2 mL of distilled water; note the changing of color indicating a leakage of Rhodamine B.

The connected implant abutments were placed into 15 mL falcon tubes previously filled with 2 mL of distilled water (Figure 3).

The vials were protected from the light and placed on a shaker for 48 hours for a homogenous distribution of Rh B in the water (150 rpm/min) (GFL 3005 Gesellschaft für Labortechnik mbH, Burgwedel, Germany).

For each group, spectrophotometric analysis was performed with a spectrophotometer (Hitachi Fluorescence Spectrophotometer, Tokyo, Japan) with a special cuvette (Perkin Elmer Luminescence Spectroscopy Cells Part number B0631104) at 1 h (T0) and 48 h (T1) at room temperature. 1 mL of those 2 mL was taken at each time (T0 and T1) to do the spectrophotometric analysis. Note that the implant-abutment set was immersed in the water all the incubation time due to the conical form of the vial.

The fluorescence present in the water and measured by the fluorometer indicates (using the calibration curve) the concentration of Rh B in this water at T0 and T1. Then by simple calculation, knowing the concentration, we determine the volume of the leak of Rh B from the inside of the implant to the water using the formula: Initial  Concentration × Initial  Volume = Final  Concentration × Final  Volume [38].

### 2.3. Statistical Analysis

Since we are dealing with differences between group means and their associated procedures, we have used a statistical test based on the analysis of variance. Unpaired student's *t*-test was also used for comparison between groups. Our results are expressed as means ± SD, and *n* refers to the number of independent samples in independent experiments. Differences were considered significant at *P* < 0.05.

## 3. Results

**Figure 4:**
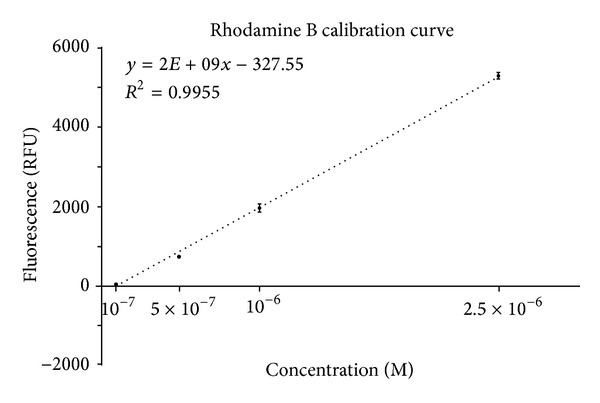
Calibration curve was determined by linear regression considering the fluorescence (*R* ^2^ 0.9955). The unit used for fluorescence was relative fluorescence of Rhodamine units (RFU).

Calibration curve was determined by linear regression considering the fluorescence (*R* ^2^ 0.9955) (Figure 4).

**Figure 5:**
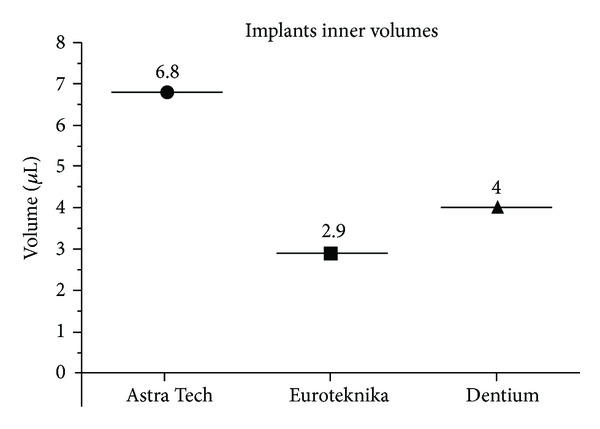
The measurements of the inner volumes were as: Astra Tech (6.8 *μ*L), Euroteknika (2.9 *μ*L), and Dentium (4 *μ*L).

The results showed that Astra Tech have the highest inner volume (6.8 *μ*L); Dentium (4 *μ*L) and Euroteknika have the smallest one (2.9 *μ*L) (Figure 5).

**Figure 6:**
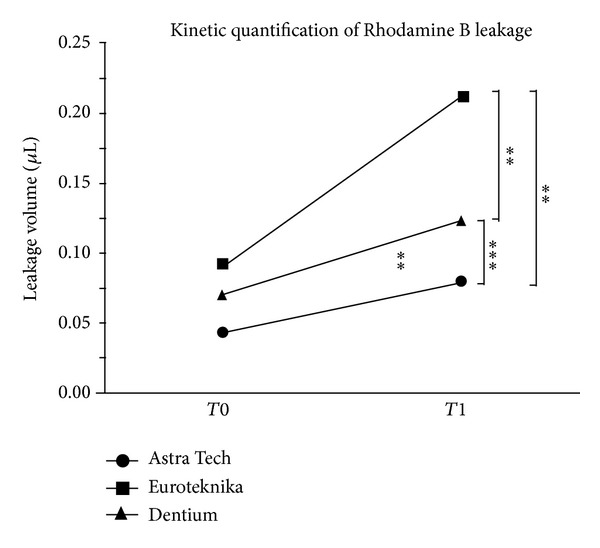
At T0 and T1, respectively, the leakage volume of each system is shown. According to the Prism convention of significance, the results were considered significant: **P ≤ 0.01; ***P ≤ 0.001.

At T0 and T1, respectively, the leakage volume and the leakage percentage of each system were as follows: Astra Tech 0.043 *μ*L or 1.48% (SD 0.0022), 0.08 *μ*L or 5.56% (SD 0.0074), Euroteknika 0.09 *μ*L or 6.93% (SD 0.0913), 0.21 *μ*L or 20.55% (SD 0.0035), and Dentium 0.07 *μ*L or 4.6% (SD 0.0029), 0.12 *μ*L or 10.47% (SD 0.0072) (Figure 6).

Using the unpaired *t*-test statistical analysis in all of the three comparisons and according to the Prism convention of significance, the results can be considered as significant: **P ≤ 0.01; ***P ≤ 0.001 (Table 1).

The statistical analyses of the leakage results are summarized in Table 2.

## 4. Discussion

Achieving peri-implant bone height in implant therapy is a challenging procedure and maintaining it over time can be an equally demanding task. Its maintenance is subject to both mechanical [5, 7, 10, 39, 40] and microbiological aspects of the implant abutment connection [1, 12, 16].

Leakage is an important factor that should be taken into consideration when choosing an implant system and its components. Thus, in vitro assessment of leakage is of primary concern.

The aim of the present study was the detection of leakage through the implant-abutment connection of three implant systems having the same interface using Rhodamine B after measuring the inner volumes.

Different techniques were used for the evaluation of leakage. Colored tracing probes [41], bacteria [2, 20–24], endotoxin, [25–27], toluidine blue [28], and gas flow method [29, 30] have all been used. As microbiological studies are in general very sensitive to handle and since biological agents are susceptible to changes in the working area, Rhodamine B was used as a tracing dye. To measure the exact exchange volume, the concentration of Rhodamine B can be calculated in a very accurate way, based on the fluorescence intensity.

The inner volume of implant abutments can vary a lot between different implant systems. Different amounts of suspension have been used to inoculate the implants in microleakage studies. Amounts of suspension range between 0.3 *μ*L [31], 0.5 *μ*L [21, 25], 0.7 *μ*L [28], 3 *μ*L [24], and 5 *μ*L [20]. In in vitro studies, the determination of the inner volume using a specific dye is mandatory prior to the evaluation of the leakage. Insufficient amount of solution or excess may lead to false positive or false negative results. The inner volumes determination was therefore determined first using a stereomicroscope and was confirmed on all seven implants of each group, at least three times. The inoculation volume of Rhodamine B was increased to 0.1 *μ*L each time to finally reach the maximal volume that shows no leak on the microscope.

Even though the three implant systems have the same prosthetic interface, the results showed a wide variation of volume between them: 6.8 *μ*L for Astra Tech, 4 *μ*L for Dentium, and 2.9 *μ*L for Euroteknika. At T1, Astra Tech and Dentium showed an increase of 4.1% and 5.9% in the leakage volume, respectively, while Euroteknika showed an increase of 13.6%.

Astra Tech showed the lowest leaking rate after 48 hours followed by Dentium, while Euroteknika presented the highest leaking rate after the same period of time.

Also as mentioned before, Astra Tech has the highest inner volume and Euroteknika has the lowest one. The inner volume seems to have no effect on the leakage. Leakage is probably affected by the close fit between the abutments and implants and the resulting gap between these components.

The choice of T1 to be 48 hours was made after trying different time points (1, 2, 4, 12, 24, 48, and 72 hours and 7 days). Interestingly, the most statistically significant measurement of leakage increase was observed at 48 hours. Moreover, this time point was sufficient to show the full kinetic evolution of the leakage, resulting in a maximum increase of the fluorescence intensity. Furthermore, the measurement of the fluorescence after 7 days did not show any significant increase in the results obtained from measurement at 48-hour time point. This result was in accordance with Harder and colleagues [25] and Aloise and colleagues [42] observations.

Regardless of the used techniques, all systems presented some degree of leakage. Jansen and colleagues [2] compared thirteen different implant-abutment combinations of different systems and concluded that even with a marginal gap less than 10 microns all implant systems presented microbial leakage. Gross and coworkers [41] found that the color marker was released through the implant-abutment gap when comparing between systems presenting external hexagons, spline, and morse taper and varied according to the brand and torque applied. Aloise and collaborators [42] showed that morse taper implant systems, Bicon and Ankylos, are unable to completely prevent bacterial leakage and colonization 48 hours after incubation. Harder and colleagues [25] have demonstrated that even internal conical implant-abutment connections were not tight on the molecular level while Astra Tech implants presented a higher tightness than ANKYLOS. Teixeira and coworkers [21] evaluated the leakage through the morse taper and internal-hexagon connection and found high degrees of leakage. Fauroux and collaborators [30] comparing leakage between four conical connection systems using gas flow showed that connection design is not the most important parameter for implant-abutment connection leakage.

In the present study of the three implant systems with the same conical connection, the accuracy in fabrication and the precision of fit of the components seem to be the most important factors to consider. By using Rhodamine B fluorescence intensity measurement for the detection of microleakage at implant-abutment interface, accurate measurements were obtained and the instability of bacterial culture in vitro was avoided.

## 5. Conclusion

Within the limitations of this in vitro study, the hypothesis that implant systems with the same prosthetic interface have the same inner volume and are similar regarding leakage was rejected. Astra Tech implants show the biggest inner volume and significantly the least microleakage compared to Dentium and Euroteknika implant systems.

## Figures and Tables

**Table 1:** It presents a summary of all the results: the mean of the leakage volumes for each system at T0 and T1, the standard deviations, and *P* values (differences were considered significant at *P* < 0.05). The inoculation volume for each system is also presented.

	Astra Tech 6.9 *μ*L			Euroteknika 3 *μ*L			Dentium 4.1 *μ*L		
	Mean	SD	*P*	Mean	SD	*P*	Mean	SD	*P*
T0	0.0432	0.0022	0.0029	0.09133	0.0035	0.0081	0.0707	0.0029	0.0005
T1	0.0792	0.0074	0.0029	0.2117	0.0129	0.0081	0.1226	0.0072	0.0005

**Table 2:** Presents the leakage volumes and percentages for each system at T0 and T1 after normalization of the results.

	T0	T1
Astra Tech 6.9 *μ*L	0.043 *μ*L	0.08 *μ*L
Dentium 4.1 *μ*L	0.07 *μ*L	0.12 *μ*L
Euroteknika 3 *μ*L	0.09 *μ*L	0.21 *μ*L

Astra Tech 6.9 *μ*L	1.48%	5.56%
Dentium 4.1 *μ*L	4.6%	10.47%
Euroteknika 3 *μ*L	6.93%	20.55%
